# The balance between AIM2-associated inflammation and autophagy: the role of CHMP2A in brain injury after cardiac arrest

**DOI:** 10.1186/s12974-021-02307-8

**Published:** 2021-11-05

**Authors:** Rongjiao Shao, Xintao Wang, Tianhua Xu, Yiyang Xia, Derong Cui

**Affiliations:** Department of Anesthesiology, Shanghai Jiao Tong University Affiliated Sixth People’s Hospital, No.600 Yishan Road, Xuhui District, Shanghai, 200233 China

**Keywords:** CHMP2A, AIM2 inflammasome, Autophagy, Inflammation, Cardiac arrest, Brain damage

## Abstract

**Background:**

Activation of the absent in melanoma 2 (AIM2) inflammasome and impaired autophagosome clearance in neurons contribute significantly to cardiac arrest and return of spontaneous circulation (CA-ROSC) injury, while the mechanism by which the AIM2 inflammasome is regulated and relationship between the processes remain poorly understood. Recently, charged multivesicular body protein 2A (CHMP2A), a subunit of endosomal sorting complex required for transport (ESCRT), was shown to regulate phagophore closure, and its depletion led to the accumulation of autophagosomes and induced cell death. Here, we investigated whether CHMP2A-mediated autophagy was an underlying mechanism of AIM2-associated inflammation after CA-ROSC and explored the potential link between the AIM2 inflammasome and autophagy under ischemic conditions.

**Methods:**

AIM2 inflammasome activation and autophagic flux in the cortex were assessed in the CA-ROSC rat model. We injected LV-Vector or LV-CHMP2A virus into the motor cortex with stereotaxic coordinates and divided the rats into four groups: Sham, CA, CA+LV-Vector, and CA+LV-CHMP2A. Neurologic deficit scores (NDSs), balance beam tests, histopathological injury of the brain, and expression of the AIM2 inflammasome and proinflammatory cytokines were analyzed.

**Results:**

AIM2 inflammasome activation and increased interleukin 1 beta (IL-1β) and IL-18 release were concurrent with reduced levels of CHMP2A-induced autophagy in CA-ROSC rat neurons. In addition, silencing CHMP2A resulted in autophagosome accumulation and decreased autophagic degradation of the AIM2 inflammasome. In parallel, a reduction in AIM2 contributed to autophagy activation and mitigated oxygen–glucose deprivation and reperfusion (OGD-Rep)-induced inflammation. Notably, CHMP2A overexpression in the cortex hindered neuroinflammation, protected against ischemic brain damage, and improved neurologic outcomes after CA.

**Conclusions:**

Our results support a potential link between autophagy and AIM2 signaling, and targeting CHMP2A may provide new insights into neuroinflammation in the early phase during CA-ROSC.

**Supplementary Information:**

The online version contains supplementary material available at 10.1186/s12974-021-02307-8.

## Background

Cardiac arrest (CA) is an acute event that challenges global public health [1]. Although return of spontaneous circulation (ROSC) after CA is considered a state of successful cardiopulmonary resuscitation, most patients with ROSC develop nervous system impairment because of CA-induced global cerebral ischemia/reperfusion injury [2]. The pathological mechanisms responsible for neuronal damage after CA-ROSC are highly complex, and the inflammatory response mediated by multiprotein complexes known as inflammasomes has been widely recognized as playing a leading role [3]. Absent in melanoma 2 (AIM2) is a member of the ALR family of proteins and senses double-stranded DNA to initiate the AIM2 inflammasome complex, which includes AIM2, apoptosis-associated speck-like protein containing a CARD (ASC), and caspase-1 [4, 5]. AIM2 inflammasomes are a common focus of ischemic stroke research. Previous work indicates that deletion or inhibition of the AIM2 inflammasome alleviates brain injury after ischemic stroke [5, 6]. However, the mechanism by which the AIM2 inflammasome is regulated remains less well understood. Interestingly, recent evidence has shown that autophagy may also regulate the AIM2 inflammasome [7].

Macroautophagy is an evolutionarily conserved process of lysosomal degradation and recycling of cellular components and is essential for maintaining neuronal homeostasis and functions [8]. Autophagy comprises five key stages: nucleation, elongation of the phagophore, autophagosome closure and maturation, autophagosome fusion with the lysosome, and selective degradation [9]. Importantly, efficient autophagic flux is vital for neuronal cell survival, while impaired autophagic flux causes neuronal dysfunction during cerebral ischemia [10]. The underlying mechanisms may be defects in the stage of autophagy, such as lysosomal dysfunction [11]. However, much less is known about the late stages of autophagy, particularly autophagosome maturation, in which the double membrane seals to enclose the contents and then fuses with the lysosome [12]. Recently, the endosomal sorting complex required for transport (ESCRT) subunit charged multivesicular body protein 2A (CHMP2A) was shown to regulate phagophore closure [13, 14]. CHMP2A depletion leads to the accumulation of autophagosomes and induces cell death [12].

The association between autophagy and inflammasomes is complex. Autophagy dysfunction leads to excessive activation of inflammasomes [15, 16]. Likewise, inflammasome signaling pathways can upregulate autophagy to inhibit detrimental responses [17, 18]. Hence, the balance of these processes plays a decisive role in the outcome of brain injury [19]. However, whether impaired autophagic flux leads to AIM2-associated inflammation and pyroptotic cell death during CA-ROSC remains unclear. In the present study, we first observed that AIM2 inflammasome activation paralleled the CHMP2A-mediated impairment of autophagic flux after exposure to CA-ROSC. Notably, we examined the relationship between impaired autophagic flux and the AIM2 inflammasome and elucidated whether CHMP2A-mediated autophagy was an underlying mechanism of neuroinflammation after CA-ROSC.

## Materials and methods

### Ethics approval

Animal care and experimental procedures were approved by the Animal Ethics Committee of the Shanghai Jiao Tong University Affiliated Sixth People’s Hospital (approval code: DWSY2020–0112). The animals were supplied by the Animal Research Committee of Shanghai Jiao Tong University [SYXK (Shanghai, China) 2016–0020]. All efforts were made to minimize any suffering or discomfort and the number of animals used in the current study.

### Rat model of CA-ROSC

The rats were anesthetized by the intraperitoneal injection of pentobarbital sodium (40 mg/kg). Next, the rats were fixed on a homeothermic board in the supine position, and their rectal temperature was maintained at 37.5 °C ± 0.5 °C. The rats were endotracheally intubated with a 14G cannula and connected to a rodent ventilator (#55704; Harvard Apparatus Model, Holliston, MA, USA). The right femoral artery and vein were intubated with a catheter (TERUMO Surflash i.v. catheter) to dynamically monitor blood pressure and for drug administration and liquid intervention. After 10 min of stabilization, asphyxia was induced by vecuronium bromide (2 mg/kg) and ventilator disconnection for 5 min, resulting in the cessation of the arterial pulse and the MAP less than 25 mmHg. After 5 min of asphyxia, cardiopulmonary resuscitation (CPR) was initiated by mechanical ventilation (100% oxygen), the intravenous application of epinephrine (0.01 mg/kg) and bicarbonate (1 mEq/kg), and concurrent finger chest compressions (approximately 200 beats per min). Additional doses of epinephrine were repeatedly injected until ROSC was achieved. Rats were omitted from further experiments if spontaneous circulation did not recover within 5 min of CPR. After successful rescue, characterized as an increase in MAP not less than 60 mmHg for more than 10 min, all care was standardized. The sham rats underwent surgery but no CA.

### Behavioral tests

Approximately 30 min before testing, all the animals were placed in a behavioral test room to acclimate to the environment. All behavioral tests were performed by investigators who were blinded to the treatment groups.

### Neurologic deficit score (NDS)

CA-ROSC rats were followed up for 21 days. Neurological functions were assessed at 24, 48, and 72 h and 7, 14, and 21 days after ROSC according to the NDS. Five parameters—arousal, cranial nerve reflexes, motor function, sensory function, and simple behavioral responses—were scored according to NDS criteria (0–80 scale; 0 brain dead, 80 best). To evaluate the level of neurologic function in rats, we predetermined the NDS cutoff points for good (NDS ≥ 60) and poor (NDS < 60) outcomes [20].

### Balance beam test

The balance beam test was performed at the same time as the NDS to assess rat motor coordination and balance [21]. The rats were trained four times daily for 3 consecutive days before surgery on an elevated (30 cm above the ground) narrow wooden beam (1.5 cm wide × 100 cm long). Following surgery, the behaviors of the rats were observed and tested according to the scoring standards listed in Table 1.

**Table 1:** Balance beam test

Description	Points
Falls off with no attempt to balance or hang on to the beam (< 20 s)	6
Attempts to balance on the beam but falls off (> 20 s)	5
Attempts to balance on the beam but falls off (> 40 s)	4
Hugs beam and two limbs fall down from the beam, or spins on the beam (> 60 s)	3
Hugs beam and one limb falls down from the beam	2
Grasps side of the beam	1
Balances with steady figure (> 60 s)	0

### Cell culture and drug administration

Highly differentiated rat pheochromocytoma PC12 cells were obtained from the Institute of Cell Biology, Chinese Academy of Sciences (Shanghai, China). The cells were cultured in Dulbecco’s modified Eagle’s medium (DMEM) containing 10% fetal bovine serum (FBS) and 1% dual antibiotics (15140–122, Gibco) and were incubated at 37 °C in an atmosphere with 5% CO_2_/95% air.

For drug administration, the indicated concentrations of lipopolysaccharide (LPS) (500 ng/mL; L3127; Sigma), rapamycin (Rapa) (5 μmol/L; ab120224; Abcam), chloroquine (CQ) (10 μmol/L; C6628; Sigma), 3-methyladenine (3-MA) (10 μmol/L; M9261; Sigma), or vehicle control were dissolved in normal medium and administered to the cells.

### OGD-Rep model and cell viability

For OGD-Rep stimulation, PC12 cells were washed twice with phosphate-buffered saline (PBS) (pH 7.4), cultured in glucose-free DMEM (11966–025; Gibco) and then immediately placed in a sealed chamber (MIC-101; Billups-Rothenburg) under hypoxic conditions (95% N_2_ and 5% CO_2_) for 6 h, after which the cells were placed under normal conditions for 3 h for reperfusion. Control PC12 cells were grown in complete medium for 6 h at 37 °C. PC12 cells were cultured in 96-well plates at a density of approximately 2 × 10^3^ cells per well under normal conditions for 24 h. After treatment, cell viability was evaluated compared with that of control cells using Cell Counting Kit-8 (CCK-8) (#CK04; Dojindo, Japan). Absorbance was measured at 450 nm after 2 h of incubation using a microplate reader (ELX800; BioTek, USA).

### Stereotactic surgery and cell transfection

The rats were deeply anesthetized with pentobarbital sodium (40 mg/kg) and placed in a stereotaxic frame (Stoelting, IL, USA). After the skull was exposed, a perpendicular hole was made to allow for injections. Lenti-*EGFP* vector or Lenti-*EGFP-CHMP2A* virus (4 × 10^8^ transducing units (TU)/mL) (Obio Technology, Shanghai, China) was injected into the motor cortex with the following stereotaxic coordinates: (a) from the bregma: AP: + 1.7 mm; ML: ± 1 mm; DL: − 2 mm; (b) from the bregma: AP: + 1.7 mm; ML: ± 2.5 mm; DL: − 2.5 mm. At each injection site, 1 μL of concentrated lentivirus was injected at a speed of 0.1 μL/min using a 10-μL Hamilton syringe with a 33 G needle (65460–05). To prevent reflux, the needle remained in place for 5 min before being withdrawn. The CA model was established 1 week after stereotactic injection, and all the off-target rats were excluded from the study.

PC12 cells (2 × 10^4^ cells/mL) were seeded in six-well plates, and lentiviruses containing CHMP2A and AIM2 were used to transfect the cells and achieve high transfection efficiency. shRNAs targeting rat CHMP2A (5′-AAGATGAAGAGGAGAGTGA-3′) and rat AIM2 (5′-GGTCACCAGTTCCTCAGTT-3′) and a scrambled control shRNA (5′-AUGAAGTGAAUUGCUCAA-3′) were purchased from Obio Technology (Shanghai, China). After 8 h of transfection in antibiotic-free medium, the cells were placed in fresh medium and transfected for an additional 72 h.

### Transmission electron microscopy (TEM)

PC12 cells were scraped gently and collected into centrifuge tubes after fixation with precooled 2.5% glutaraldehyde. Next, the cells were centrifuged at 1000 r/min for 5 min until the cell masses were visible. Fresh glutaraldehyde was then added. Next, the samples were fixed with 1% osmium tetroxide, dehydrated in graded ethanol, and embedded in Epon 812. Autophagosomes were observed using an HT7800 transmission electron microscope (Hitachi, Tokyo, Japan).

### Animals

Healthy male adult Sprague–Dawley rats (300–400 g) were housed in a specific pathogen-free facility with ad libitum access to food and water under a strict 12-h light/dark cycle.

### Nissl staining

Nissl staining was performed according to the manufacturer’s instructions (C0117; Beyotime Technology, China). Briefly, coronal sections (10 μm) were soaked in Nissl staining solution for 10 min at 37 °C and quickly washed with double-distilled water. Next, the sections were dehydrated with gradient ethanol and cleared in xylene for 2 min. Damaged neurons were characterized by shrunken nuclei and condensed staining, while normal neurons showed regular cell morphology and round nuclei [22]. Five different regions of each group were used for quantification, and the mean number of surviving neurons was measured using ImageJ software (version 1.8.0; NIH, Bethesda, MD, USA).

### Immunofluorescence staining

At designated times after CA-ROSC, the rats were anesthetized, perfused transcardially with ice-cold saline, and fixed with 4% paraformaldehyde (PFA) (in 0.1 M PB, pH 7.4). The brains were quickly removed, postfixed in 4% PFA for 24 h at 4 °C and then dehydrated in 20%/30% sucrose. After embedding in optimum cutting temperature compound (#4583; SAKURA), the brains were serially cut into 10 μm frozen sections and mounted on slides. The brain tissue cryosections were blocked with 10% goat serum (G9023; Sigma Aldrich) in Tris-buffered saline (TBS) containing 0.3% Triton X-100 (X100; Sigma Aldrich) for 1 h at room temperature. Next, the slides were incubated with primary antibodies overnight at 4 °C and further incubated with secondary antibodies for 2 h at room temperature. Subsequently, cell nuclei were stained with DAPI (0100–20; Southern Biotech) for 5 min, followed by observation and photography.

PC12 cells grown on glass coverslips (#801007; NEST Biotechnology, China) were fixed in 4% PFA for 20 min and permeabilized with 0.1% Triton X-100 for 15 min. Next, the cells were blocked with 10% goat serum for 30 min. After incubation overnight with primary antibodies, cells on the coverslips were washed three times with PBS and incubated with secondary antibodies for 30 min in the dark. Cell nuclei were stained with DAPI for 5 min at room temperature.

The primary antibodies used for immunochemistry included antibodies against NeuN (1:100; 94403S; Cell Signaling Technology), GFAP (1:100; ET1601–23; HuaBio, China), Iba1 (1:500; 019–19741; Wako, Japan), LC3 (1:100; NB100–2220; Novus Biologicals), SQSTM1/p62 (1:200; ab56416; Abcam), CHMP2A (1:200; 10477–1-AP; Proteintech), GSDMD (1:200; 20770–1-AP; Proteintech), Caspase-1 (1:200; sc-398715; Santa Cruz), AIM2 (1:200; sc-293174; Santa Cruz), IL-1β (1:100; sc-515598; Santa Cruz) and IL-18 (1:200; 10663–1-AP; Proteintech). Goat anti-mouse and goat anti-rabbit Alexa 546- or Alexa 488-conjugated antibodies were used as secondary antibodies. Microscopy images were obtained using a Leica DM IL LED inverted microscope (Buffalo Grove, IL, USA) and a Zeiss LSM 710 confocal microscope (Athens, GA, USA). The images were analyzed using ImageJ software (version 1.8.0; NIH, Bethesda, MD, USA).

### Western blot analysis

Total protein was extracted from PC12 cells and brain tissues. The protein concentrations of these samples were determined using a BSA kit (P0009; Beyotime Technology) according to the manufacturer’s instructions. Equal amounts of protein (15–90 μg) were loaded onto 12.5% SDS–polyacrylamide gels and separated using a Tris–glycine system. Next, the protein bands were transferred to 0.22 μm or 0.45 μm PVDF membranes (Millipore, Boston, MA, USA), which were blocked with 5% nonfat-powdered milk in TBS plus 0.1% Tween (TBST) for 90 min at room temperature. The membranes were then incubated with appropriate primary antibodies (listed below) at 4 °C with gentle shaking, followed by incubation with secondary antibodies for 1 h at room temperature. Bound proteins were detected using ImageQuant LAS 4000 (Amersham Imager 600; General Electric Company, USA) with enhanced chemiluminescence (CPS160; Millipore, USA). For quantification, the band densities were determined using ImageJ software (version 1.8.0; NIH, Bethesda, MD, USA).

The following primary antibodies were used for immunoblotting: mouse anti-AIM2 (1:1000; sc-293174; Santa Cruz), mouse anti-caspase-1 (1:500; sc-398715; Santa Cruz), mouse anti-ASC (1:500; sc-514414; Santa Cruz), mouse anti-CHMP3 (1:1000; sc-166361; Santa Cruz), mouse anti-CHMP7 (1:1000; sc-271805; Santa Cruz), mouse anti-CEP55 (1:1000; sc-374051; Santa Cruz), rabbit anti-CHMP2A (1:1000; 10477–1-AP; Proteintech), rabbit anti-CHMP2B (1:1000; #76173S; Cell Signaling Technology), rabbit anti-LC3 (1:1000; NB100–2220; Novus Biologicals), mouse anti-p62 (1:1000; ab56416; Abcam), rabbit anti-Beclin-1 (1:1000; D40C5; Cell Signaling Technology), rat anti-IL-18 (1:1000; 10663-1-AP; Proteintech), mouse anti-IL-1β (1:500; sc-515598; Santa Cruz), rabbit anti-GSDMD (1:1000; 20770-1-AP; Proteintech), and mouse anti-β-actin (1:10000; M1210-2; Huabio). The secondary antibodies were goat anti-rabbit (1:5000; HA1001–100; Huabio) and goat anti-mouse (1:10000; HA1006; Huabio).

### Quantitative real-time PCR

Total RNA was extracted from cortical tissue and cells using a commercial RNA isolation kit (RC112; Vazyme). For RT-PCR analysis, cDNA synthesis was performed using the ABScript II RT Mix for qPCR (RK20402; ABclonal), and quantitative PCR analyses were performed using 2× SYBR Green Fast qPCR Mix (RK21204; ABclonal) in an ABI QuantStudio7 Flex Real-Time PCR System (Applied Biosystems, USA). All relative gene expression levels were normalized to β-actin expression and calculated using the 2^−ΔΔ*Ct*^ method. All the primers used in this study are listed in Table 2.

**Table 2:** Primer sequences

Species	Gene	Sepuence
Rat	AIM2	5′-TGTTCTGTTGCTTCTGCCACCTG-3′
		5′-GCTTCCTGTTCTGCCACCATCTG-3′
Rat	NLRP3	5′-GCTGAACTTGAGCAACAACG-3′
		5′-CACCCAACTGTAGGCTCTGC-3′
Rat	NLRP1	5′-GGGGCAGCCAAATCAAGTTC-3′
		5′-TGAGCGGTCATTGCAACTCT-3′
Rat	NLRC4	5′-GGCTGAGGCCCACGTATAAA-3′
		5′-CTCCTCTGGCTCTCTGGACT-3′
Rat	P62	5′-GCTGCCCTGTACCCACATCT-3′
		5′-CGCCTTCATCCGAGAAAC-3′
Rat	LC3	5′-TTTGTAAGGGCGGTTCTGAC-3′
		5′-CAGGTAGCAGGAAGCAGAGG-3′
Rat	IL-18	5′-GCCTGTGTTCGAGGATATGACT-3′
		5′-ACCACTTTGGCAGACTTCACT-3′
Rat	IL-1β	5′-TGGCAACTGTCCCTGAACTC-3′
		5′-GTCGAGATGCTGCTGTGAGA-3′
Rat	β-actin	5′-CATCCTGCGTCTGGACCTGG-3′
		5′-TAATGTCACGCACGATTTCC-3′

### Statistical analysis

All the data are presented as means ± SD, and GraphPad Prism 8.4 software (GraphPad Software, Inc., La Jolla, CA, USA) was used for statistical analysis and graph layouts. Differences between groups were analyzed by unpaired Student’s *t* test (for single comparisons) or one-way ANOVA followed by Tukey’s post hoc tests (for multiple comparisons). NDSs were examined by two-factor (group × time) repeated-measures ANOVA. *P* values < 0.05 were considered statistically significant.

## Results

### AIM2 inflammasome activation synchronizes with the upregulation of inflammatory factors in the cortex after CA

**Fig. 1:**
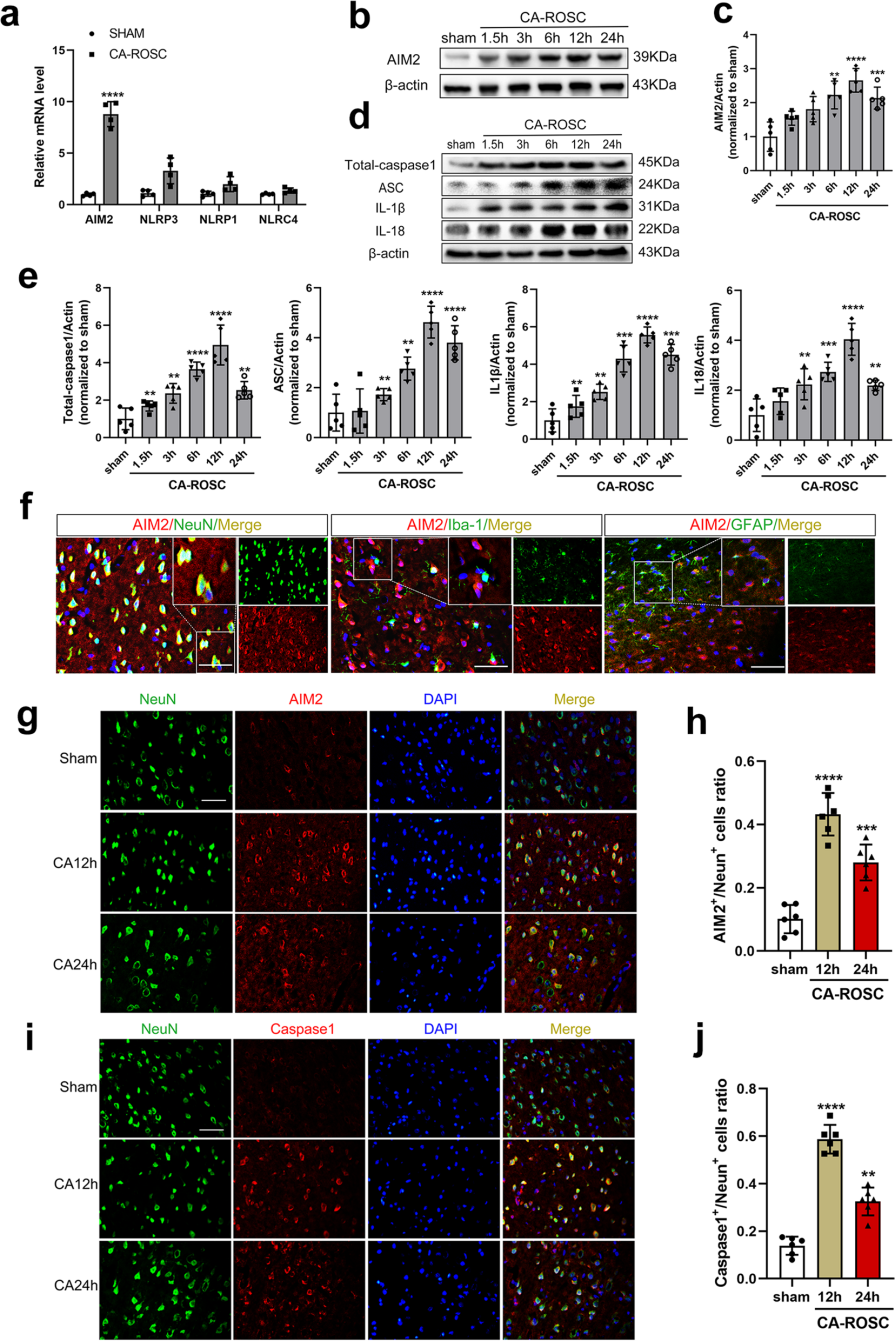
Expression levels of inflammasome components in the cortex after CA-ROSC. **a** Transcript levels of the inflammasome sensor genes AIM2, NLRP3, NLRP1, and NLRC4 in cortical tissues from sham and CA-ROSC animals. *n* = 4 per group. The data are presented as means ± SEM, Student’s *t* test. *P < 0.05 and ****P < 0.0001 vs. sham. **b**, **c** Representative Western blot and quantification of AIM2 in cortical tissue lysates from sham rats and at the indicated timepoints (1.5, 3, 6, 12, and 24 h) after CA. **d**, **e** Representative Western blot and quantification of total caspase-1, ASC, IL-1β, and IL-18 protein levels in cortical tissue lysates of sham rats and at the indicated timepoints (1.5, 3, 6, 12, and 24 h) after CA. **f** Colocalization of AIM2 (red) with neurons (NeuN, green), microglia (Iba-1, green), and astrocytes (GFAP, green) in the cortex 12 h after CA. Scale bar, 50 μm. **g**, **h** Representative immunofluorescence images and quantification of AIM2 and NeuN double staining. **i**, **j** Representative immunofluorescence images and quantification of caspase-1 and NeuN double staining. Scale bar, 50 μm. The data are expressed as means ± SEM (*n* = 5), one-way ANOVA. **P < 0.01, ***P < 0.001, and ****P < 0.0001 vs. sham

To identify the role of inflammasomes in CA-ROSC, we first evaluated the temporal expression of inflammasome sensor genes (i.e., NLRP1, NLRP3, NLRC4 and AIM2 inflammasomes) in the brains of rats subjected to CA. AIM2 (*P* < 0.0001) expression levels significantly increased more than those of NLRP1, NLRP3, and NLRC4 (Fig. 1a). In addition, further examination of AIM2 protein expression was performed by Western blotting, which revealed that the level peaked by 12 h (*P* < 0.0001) and gradually decreased by 24 h (*P* = 0.0002) after CA-ROSC (Fig. 1b, c). AIM2 is a dominant player in sterile inflammation in response to cytosolic DNA, initiating the assembly of the AIM2 inflammasome complex [23, 24]. Subsequently, the recruitment of pro-caspase-1 to the inflammasome complex leads to the processing of caspase-1, pro-IL-1β, pro-IL-18, and gasdermin D (GSDMD) [4, 5]. Therefore, we investigated the expression of other inflammasome subunits, such as ASC, caspase-1, and downstream proinflammatory cytokine precursors of both IL-1β and IL-18. These factors were also time-dependently upregulated. Between 6 and 24 h after CA-ROSC, the levels of the inflammasome components in the cortex of CA rats were higher than those in sham rats (Fig. 1d, e). Collectively, these results suggest that the AIM2 inflammasome is activated and is a risk factor in the rat model of CA-ROSC.

### The AIM2 inflammasome is mainly expressed in neurons and causes pyroptotic cell death in CA-ROSC rats

**Fig. 2:**
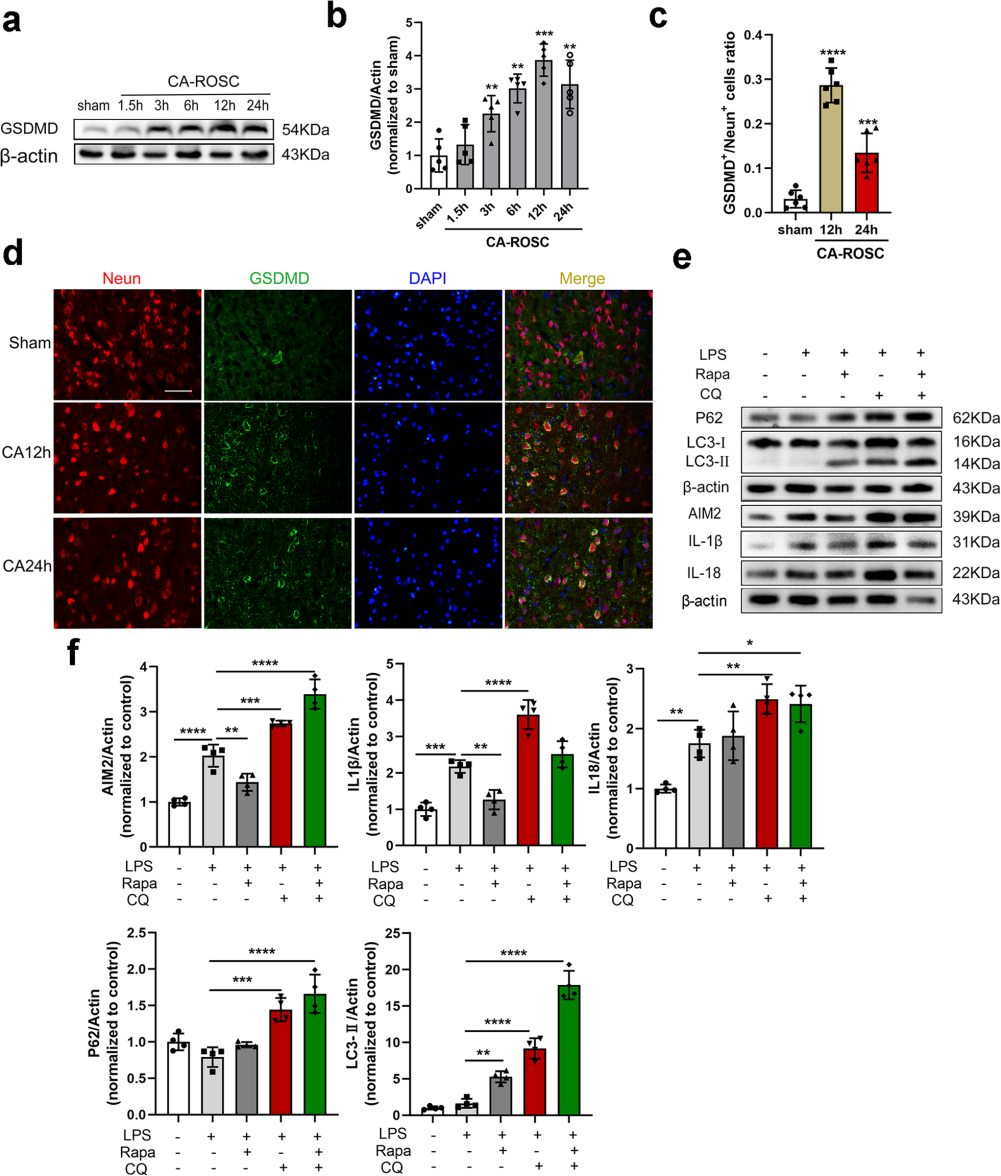
Modulating autophagy affects the AIM2 inflammasome. **a**, **b** Representative Western blot and quantification of GSDMD in the cortical tissue lysates of sham and CA-ROSC rats at the indicated timepoints. **c**, **d** Representative immunofluorescence images and quantification of GSDMD and NeuN double-staining. Scale bar, 50 μm. **e** Western blot analysis of LC3, p62, AIM2, IL-1β, and IL-18 in PC12 cells stimulated with or without LPS (500 ng/mL) for 8 h and then treated with Rapa (5 μmol/L), CQ (10 μmol/L), Rapa (5 μmol/L) plus CQ (10 μmol/L), or vehicle control for 24 h. **f** Quantification of LC3, p62, AIM2, IL-1β, and IL-18 in PC12 cell lysates. The data are expressed as means ± SEM (*n* = 3–6), one-way ANOVA. **P < 0.01, ***P < 0.001, and ****P < 0.0001

To further confirm the specific expression of AIM2 in CA-ROSC rats, we performed immunofluorescence staining for neuronal nuclei (NeuN, a marker of neurons), glial fibrillary acidic protein (GFAP, a marker of astroglia), and ionized calcium binding adaptor molecule 1 (Iba1, a marker of microglia) in the cortex. CA-induced AIM2 mostly colocalized with neurons but not microglia or astroglia (Fig. 1f). The numbers of AIM2-positive neurons and caspase-1-positive neurons were increased in the vulnerable motor cortex at 12 h after CA (both *P* < 0.0001), a finding that is consistent with the immunoblot data (Fig. 1g–j). Caspase-1 plays an essential role in AIM2 activation, cleaves proinflammatory cytokines (IL-1β and IL-18) and triggers pyroptosis (inflammatory programmed cell death) [4]. To determine whether neuronal death was caused by pyroptosis, which is characterized by swelling, membrane pore formation, and the secretion of proinflammatory cytokines [25], we analyzed GSDMD (a pore-forming protein) as a pyroptosis marker in the cortex of rats with stroke. The GSDMD protein levels peaked 12 h after CA-ROSC (*P* < 0.0001; Fig. 2a, b). These results are consistent with the increase in GSDMD-positive neurons at 12 h after CA-ROSC (*P* < 0.0001; Fig. 2c, d). Taken together, these data suggest that CA-ROSC is related to the activation of the neuronal AIM2 inflammasome, along with pyroptotic cell death.

### AIM2 inflammasome induction is affected by autophagy in vitro

Previous studies have shown that autophagy may also regulate inflammasomes and augment IL-1β production and release, suggesting that autophagy may also regulate AIM2-associated inflammation in CA-ROSC [17, 26, 27]. However, the relationship between inflammasomes and autophagy may be complex. To better understand whether the induction of AIM2 inflammasomes was affected by autophagy, we stimulated PC12 cells with LPS to analyze inflammasome activation and then treated the cells with Rapa (an agonist of autophagosome formation) and CQ (an inhibitor of autophagosome processing). LPS stimulated AIM2 (*P* < 0.0001), IL-1β (*P* = 0.0002) and IL-18 (*P* = 0.0060) levels in neurons but did not alter autophagic flux (LC3-II: *P* = 0.9136; p62: *P* = 0.3000) (Fig. 2e, f). Rapa also induced an increase in LC3-II abundance (*P* = 0.0004; Fig. 2e, f), suggesting the stimulation of autophagy, clearance of p62, and complete autophagic flux compared with those of the LPS groups (Fig. 2e, f). In addition, augmenting autophagy with Rapa decreased AIM2 inflammasome activity and IL-1β production compared with the LPS groups (*P* = 0.0084, *P* = 0.0002, respectively) (Fig. 2e, f). By contrast, blocking autophagy with CQ caused the accumulation of LC3-II (*P* < 0.0001) and p62 (*P* = 0.0002) and increased AIM2 inflammasome activity (*P* = 0.0008), as indicated by increased levels of IL-1β (*P* < 0.0001) and IL-18 (*P* = 0.0069) compared with those of the LPS groups (Fig. 2e, f). These results indicate that AIM2 inflammasome activation and IL-1β/IL-18 production are regulated by autophagic processing following pharmacologic stimulation or inhibition of autophagy.

### Increased AIM2-associated inflammation is associated with reduced CHMP2A levels and autophagy impairment in the cortex after CA-ROSC

**Fig. 3:**
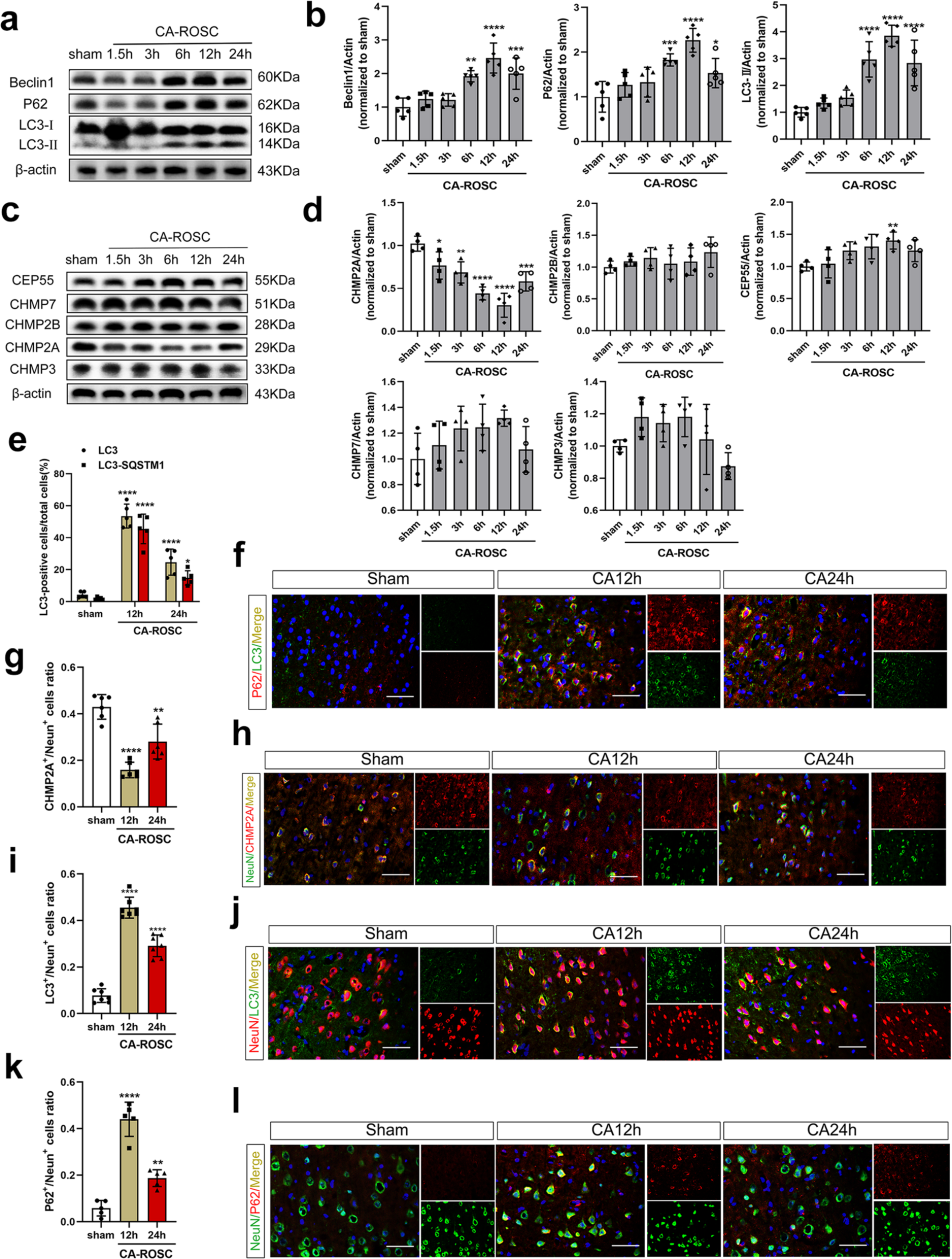
Impaired autophagic flux and expression levels of ESCRT-III components in cortical neurons after CA. **a** Representative Western blot showing Beclin-1, SQSTM1/p62 and LC3 in the cortical tissue lysates of sham and CA-ROSC rats at the indicated timepoints. **b** Quantification of Beclin-1, SQSTM1/p62 and LC3 with respect to the loading control β-actin. **c** Representative Western blot showing CHMP2A, CHMP2B, CHMP3, CEP55 and CHMP7 in the cortical tissue lysates of sham and CA-ROSC rats at the indicated timepoints. **d** Quantification of ESCRT-III components with respect to the loading control β-actin. **e**, **f** Representative immunofluorescence images of SQSTM1/p62 and LC3 double-staining and the quantification of LC3-positive cells (yellow bars) and LC3 and SQSTM1/p62 double-positive cells (red bars) in the cortex of sham-operated and injured rats. **g**, **h** Representative immunofluorescence images and the quantification of CHMP2A and NeuN double-staining. Scale bar, 50 μm. **i**, **j** Representative immunofluorescence images and quantification of LC3 and NeuN double staining. **k**, **l** Representative immunofluorescence images and quantification of SQSTM1/p62 and NeuN double staining. Scale bar, 50 μm. The data are expressed as means ± SEM (*n* = 4–6), one-way ANOVA. *P < 0.05, **P < 0.01, ***P < 0.001, and ****P < 0.0001 vs. sham

To clarify the relationship between AIM2 inflammasome activity and autophagy in CA-ROSC, we first examined the autophagy marker protein LC3-II by immunoblotting to evaluate autophagy in our model. The transformation of LC3-I to LC3-II is correlated with the extent of autophagosome formation [28]. We observed that the LC3-II protein level peaked between 6 and 12 h after CA-ROSC (both *P* < 0.0001) (Fig. 3a, b). Within 12 h of CA-ROSC, the number of LC3-positive neurons in the cortex was higher than that in sham rats (*P* < 0.0001; Fig. 3i, j). The Beclin-1 level was markedly increased in injured rats, as reflected by increased p62 protein levels (both *P* < 0.0001) (Fig. 3a, b). Beclin-1 represents the nucleation of autophagic vesicles, while p62 acts as a substrate that links LC3-II with ubiquitinated proteins for clearance during autophagy. These data suggest a higher rate of autophagy initiation and autophagosome vesicle formation but a lower resolution of autophagic events. The immunohistochemical data showed significantly more p62-positive neurons and p62-LC3 colocalization in the cortexes of injured rats as compared to the sham cortex (both *P* < 0.0001) (Fig. 3e, f, k and l). Collectively, these findings suggest that autophagic flux in cortical neurons is impaired after CA-ROSC. Notably, the peak time of impaired autophagy is consistent with AIM2 inflammasome activation.

We hypothesized three mechanisms of impaired autophagic flux in cortical neurons: (1) aberrant autophagy stimulation leads to excessive autophagosome accumulation, which blocks the clearance of cytoplasmic proteins and organelles; (2) abnormal closure of the double membrane impairs autophagosome maturation; (3) dysfunction of the autolysosome degradation pathway occurs. In our previous study [29], silencing Beclin-1 or overexpressing lysosomal-associated membrane protein 2 could not fully restore the impaired autophagic flux. Thus, we focused on autophagosome closure. According to Zhou et al., the ESCRT machinery directly regulates autophagy and catalyzes autophagosome closure [30]. Importantly, functional autolysosome formation, including lysosome recruitment and fusion, requires membrane closure [13]. Therefore, we next measured the ESCRT-III proteins CHMP2A, CHMP2B, CHMP3, CEP55, and CHMP7 in the cortex of injured and sham animals by immunoblotting [13]. The immunoblot data revealed that the level of CHMP2A significantly decreased after CA-ROSC and reached a minimum at 12 h (*P* < 0.0001; Fig. 3c, d). Consistent with the screening results, the immunofluorescence data showed a significant decrease in the number of CHMP2A-positive neurons in the injured cortex as compared to the sham cortex (*P* < 0.0001; Fig. 3g, h). These results suggest that the reduction in CHMP2A may play a role in the impairment of autophagic flux. We also assessed the colocalization of AIM2 with LC3 and AIM2 with CHMP2A 12 h after CA-ROSC (Additional file 1: Fig. S1a–d). Our results showed that AIM2 inflammasome activation is highly associated with reduced CHMP2A levels and autophagy impairment after CA-ROSC.

### Reduced CHMP2A levels induce autophagosome accumulation, while CHMP2A overexpression restores autophagic flux

Several studies have shown that ESCRT-III plays an indispensable role in basal and induced autophagy [12, 13, 30]. In addition, the depletion of CHMP2A caused the blockade and accumulation of immature autophagosomal structures [13, 31]. However, whether the observed CA-ROSC-induced reduction in CHMP2A levels modulates autophagic flux is unknown. First, we subjected PC12 cells to OGD-Rep and assessed the expression of the autophagic markers LC3-II/p62 at specific timepoints. Quantitative analysis showed impaired autophagic flux after OGD-Rep, with maximal impairment at 6 h/3 h (LC3-II: *P* < 0.0001 and p62: *P* < 0.0001) (Additional file 2: Fig. S2a and b). Overall, these data indicate impaired autophagic flux following OGD-Rep.

**Fig. 4:**
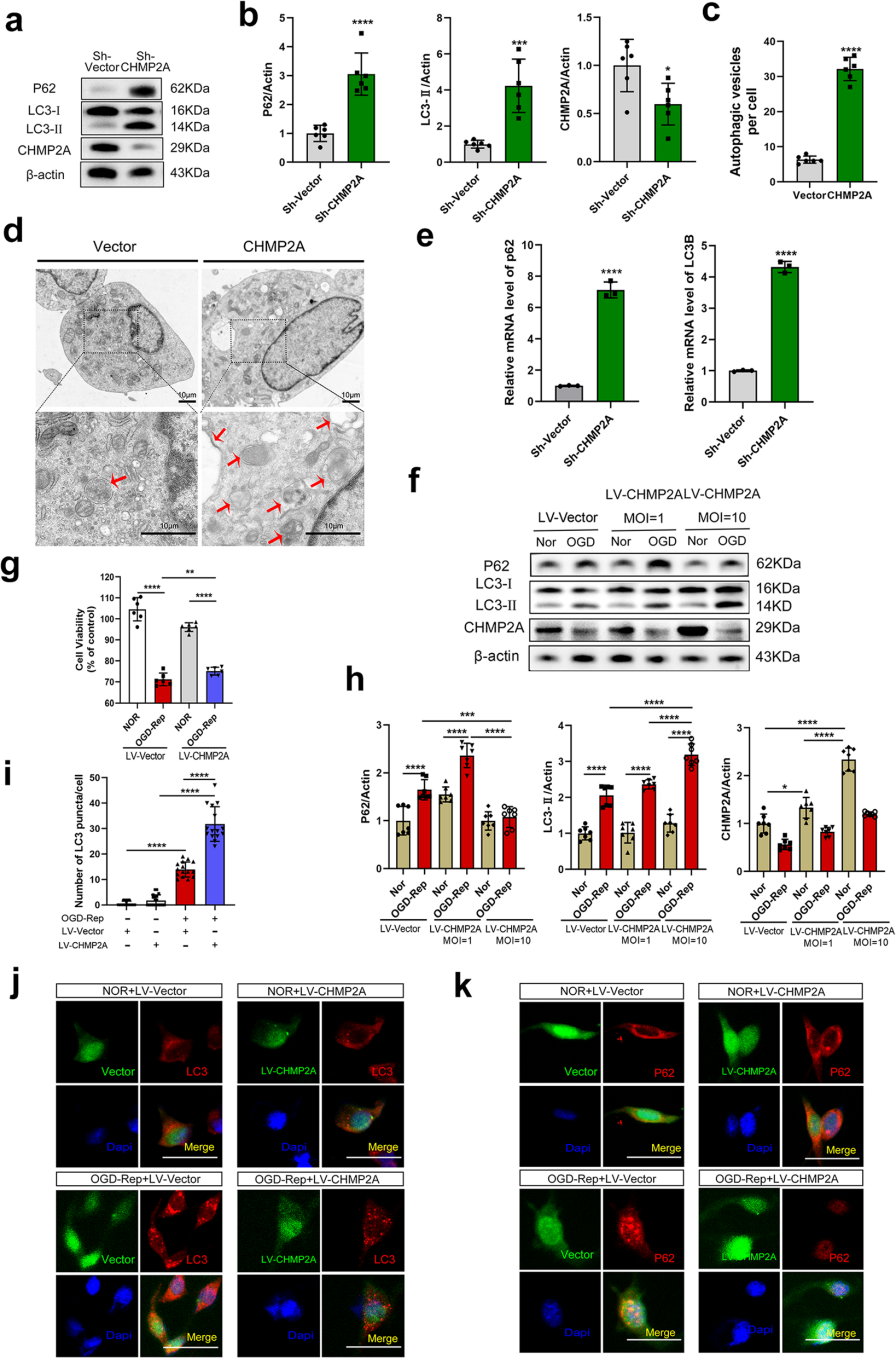
Effect of CHMP2A on autophagy in PC12 cells in vitro. **a**, **b** Representative Western blot and quantification of p62, LC3, and CHMP2A expression in PC12 cells infected with CHMP2A-specific shRNA or vector shRNA for 72 h. **c** Representative TEM analyses of autophagy in CHMP2A-silenced PC12 cells. (*n* = 6, Student’s *t* test. *P < 0.05, ***P < 0.001, ****P < 0.0001 vs. sh-vector group) **d** Autophagosome accumulation in CHMP2A-knockdown PC12 cells as shown by TEM. Red arrows indicate autophagosomes. Scale bar, 10 μm. **e** Representative RT-qPCR analyses of LC3 and p62 in PC12 cells transduced with CHMP2A or vector (20 MOI) (*n* = 3, Student’s *t* test. ****P < 0.0001 vs. sh-vector group). **f** PC12 cells were transfected with increasing doses of CHMP2A constructs (for 72 h) before OGD-Rep and were subjected to OGD or normoxia (Nor) as a control. Representative Western blot showing p62, LC3, and CHMP2A expression in PC12 cell lysates. **g** PC12 cells were transfected with CHMP2A constructs for 72 h and then were subjected to OGD-Rep. Cell viability was determined using the CCK-8 assay at 2 h (*P < 0.05, **P < 0.01, ***P < 0.001, and ****P < 0.0001). **h** Quantification of p62, LC3, and CHMP2A expression in PC12 cell lysates. **i** Quantitative analysis of LC3B puncta per cell (n > 25). **j** Representative immunofluorescence images of LC3 (red) and DAPI (blue) double-staining in PC12 cells transfected with Lenti-*EGFP* vector or Lenti-*EGFP*-*CHMP2A* virus before OGD-Rep. Puncta correspond to phagophores and/or autophagosomes. Scale bar, 50 μm. **k** Representative immunofluorescence images of p62 (red) and DAPI (blue) double-staining in PC12 cells transfected with Lenti-*EGFP* vector or Lenti-*EGFP*-*CHMP2A* virus before OGD-Rep. The data are expressed as means ± SEM (*n* = 3–6), one-way ANOVA. *P < 0.05, **P < 0.01, ***P < 0.001, and ****P < 0.0001

Next, we knocked down CHMP2A in PC12 cells with specific shRNAs. Western blot analysis showed that silencing CHMP2A hindered autophagosome formation in PC12 cells, as indicated by increases in LC3-II (*P* = 0.0003) and p62 (*P* < 0.0001) (Fig. 4a, b). Consistently, the TEM results showed that silencing CHMP2A induced autophagosome accumulation (25.83 ± 1.416; *P* < 0.0001) (Fig. 4c, d). Notably, the relative mRNA levels of LC3 and p62 in CHMP2A-silenced cells were much higher than those in vector-transfected cells (both *P* < 0.0001) (Fig. 4e). Increased p62 expression may be caused by autophagy induction. These data suggest that reduced CHMP2A protein levels induce autophagosome accumulation and suppress autophagosome clearance via the transcriptional upregulation of autophagy-related genes.

However, whether CHMP2A overexpression restores autophagic flux is unclear. Thus, we transfected PC12 cells with lentivirus to overexpress CHMP2A and assessed autophagosome formation after exposure to OGD-Rep. As shown by immunoblot analysis, minimal CHMP2A overexpression (MOI = 1) could not eliminate the impairment of autophagic flux, as indicated by the increased levels of LC3-II and p62 (both *P* < 0.0001), but CHMP2A overexpression (MOI = 10) specifically increased the abundance of LC3-II (*P* < 0.0001), indicating the restoration of autophagic flux (Fig. 4f, h). Similar to the immunoblotting results, the immunofluorescence results showed that after exposure to OGD-Rep, CHMP2A-overexpressing PC12 cells demonstrated increased LC3 puncta formation (17.87 ± 1.914 puncta/cell; *P* < 0.0001) and a dramatic decrease in p62 (Fig. 4i–k), indicating autophagosome formation and normal elimination. Interestingly, further overexpression of CHMP2A caused a significant increase in cell viability after OGD (*P* = 0.0015; Fig. 4g). These findings suggest that dose-dependent CHMP2A overexpression restores impaired autophagosomes after OGD-Rep and increases the survival of neurons.

### CHMP2A-mediated autophagy inhibits OGD-Rep-induced AIM2 inflammasome activation

**Fig. 5:**
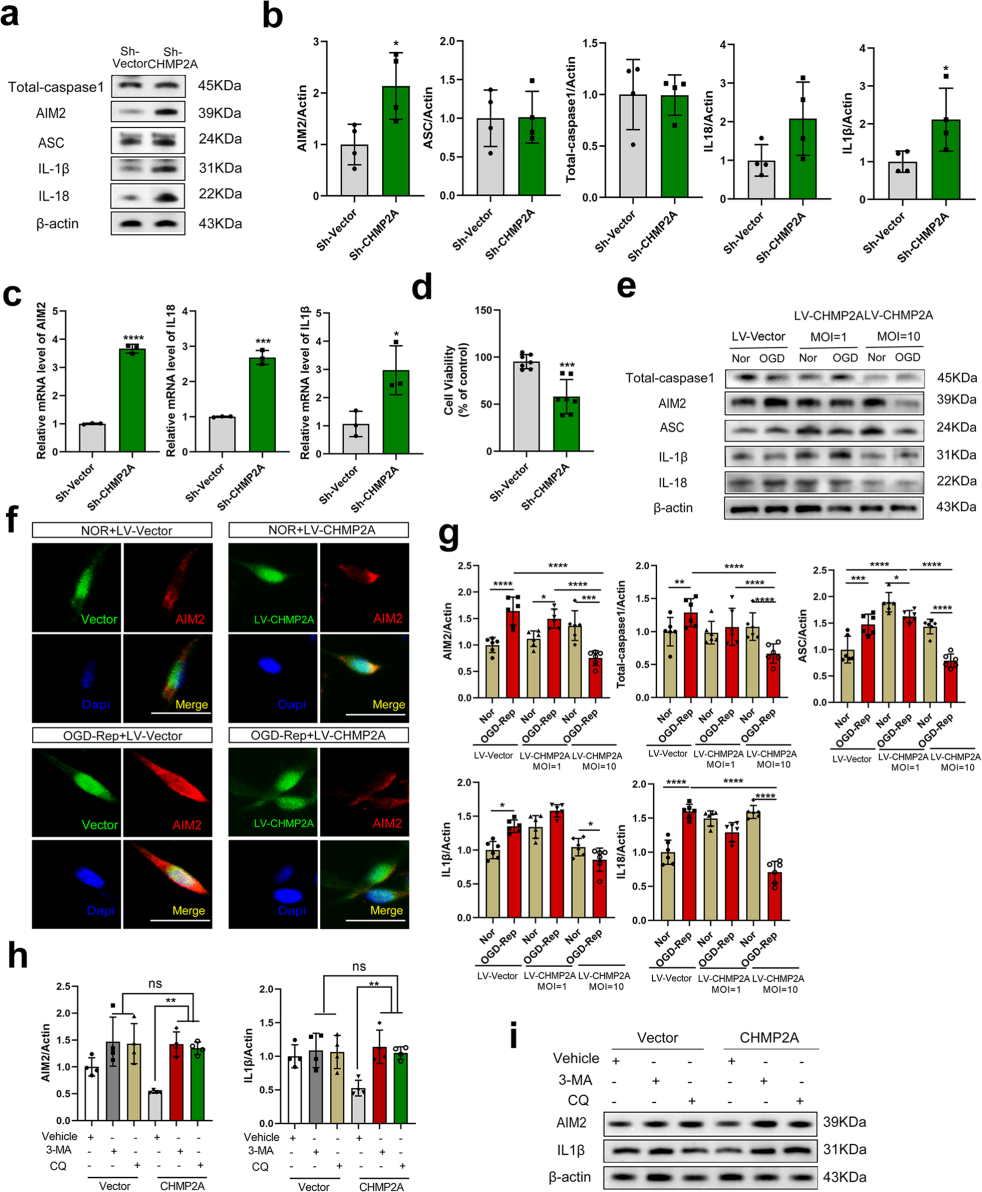
Effect of CHMP2A on OGD-Rep-induced inflammation and cell death. **a** Representative Western blot showing total caspase-1, AIM2, ASC, IL-1β, and IL-18 protein levels in PC12 cells infected with CHMP2A-specific shRNA or vector shRNA for 72 h and then subjected to OGD. **b** Quantification of total caspase-1, AIM2, ASC, IL-1β, and IL-18 protein levels in PC12 cell lysates (*n* = 4, Student’s *t* test. *P < 0.05 vs. sh-vector group). **c** Representative RT-qPCR analyses of AIM2, IL-1β, and IL-18 in PC12 cells transduced with CHMP2A or vector (20 MOI) (*n* = 3, Student’s *t* test. *P < 0.05, ***P < 0.001, and ****P < 0.0001 vs. sh-vector group). **d** Cell viability was determined by the CCK-8 assay after 2 h (***P < 0.001 vs. sh-vector group). **e** PC12 cells were transfected with increasing doses of CHMP2A constructs (for 72 h) before OGD-Rep and were subjected to OGD or normoxia (Nor) as a control. Representative Western blot showing total caspase-1, AIM2, ASC, IL-1β, and IL-18 protein levels in PC12 cell lysates. **f** Representative immunofluorescence images of AIM2 (red) and DAPI (blue) double-staining in PC12 cells transfected with Lenti-*EGFP* vector or Lenti-*EGFP*-*CHMP2A* virus before OGD-Rep. Scale bar, 50 μm. **g** Quantification of total caspase-1, AIM2, ASC, IL-1β, and IL-18 protein levels in PC12 cell lysates. **h**, **i** PC12 cells were pretreated with 3-MA (10 μM) or CQ (10 μM) before OGD-Rep. Representative Western blot and quantification of AIM2, IL-1β, and IL-18 in PC12 cell lysates (*n* = 4, one-way ANOVA. **P < 0.01)

Accumulating evidence indicates reciprocal regulation between the AIM2 inflammasome and autophagy pathways, including a defined mechanism of AIM2 degradation in a p62-dependent manner and IL-1β secretion in an LC3-dependent manner [7, 32]. To further examine the role of CHMP2A in OGD-Rep-induced AIM2 inflammasome activation, we first examined the effect of CHMP2A overexpression on AIM2 inflammasome activation after OGD. Interestingly, in OGD-primed PC12 cells, CHMP2A overexpression reduced AIM2 expression in a dose-dependent manner. A high CHMP2A expression level (MOI = 10) inhibited the expression of AIM2 (*P* < 0.0001), caspase-1 (*P* < 0.0001), and ASC (*P* < 0.0001) and the release of IL-1β (*P* = 0.0456) and IL-18 (*P* < 0.0001) compared with those of cells in the OGD-Rep group, while minimal CHMP2A overexpression (MOI = 1) did not (Fig. 5e, g). Furthermore, the immunofluorescence data showed that CHMP2A overexpression abolished OGD-induced activation of the AIM2 inflammasome (Fig. 5f). In addition, from the perspective of negative regulation, silencing CHMP2A exacerbated AIM2 inflammasome production (*t* = 3.005; *P* = 0.0239) and increased the release of IL-1β (t = 3.005; *P* = 0.0446) and cell death (*t* = 4.984; *P* = 0.0003) after OGD-Rep (Fig. 5a, b and d). Consistently, we observed significant increases in the mRNA levels of AIM2, IL-1β, and IL-18 compared with those in the vector groups (*P* < 0.0001, *P* = 0.0278, and *P* = 0.0001, respectively) (Fig. 5c). Collectively, these results further demonstrate the important role of CHMP2A in regulating the AIM2 inflammatory pathway.

To further investigate whether CHMP2A regulates the AIM2 inflammatory pathway through autophagy, we used two different autophagy inhibitors, 3-MA and CQ, which block the initial and late stages of autophagy, respectively [33], and confirmed whether autophagy inhibition could reverse the CHMP2A-mediated effects after OGD exposure. 3-MA or CQ reversed the effects of CHMP2A overexpression, leading to significant increases in the protein levels of AIM2 (*P* = 0.0041) and IL-1β (*P* = 0.0021) after OGD-Rep compared with those in the control group (Fig. 5h, i). These findings verified our hypothesis that CHMP2A-mediated autophagy inhibits OGD-Rep-induced AIM2 inflammasome activation.

### A reduction in AIM2 contributes to autophagy activation and inhibits IL-1β release

**Fig. 6:**
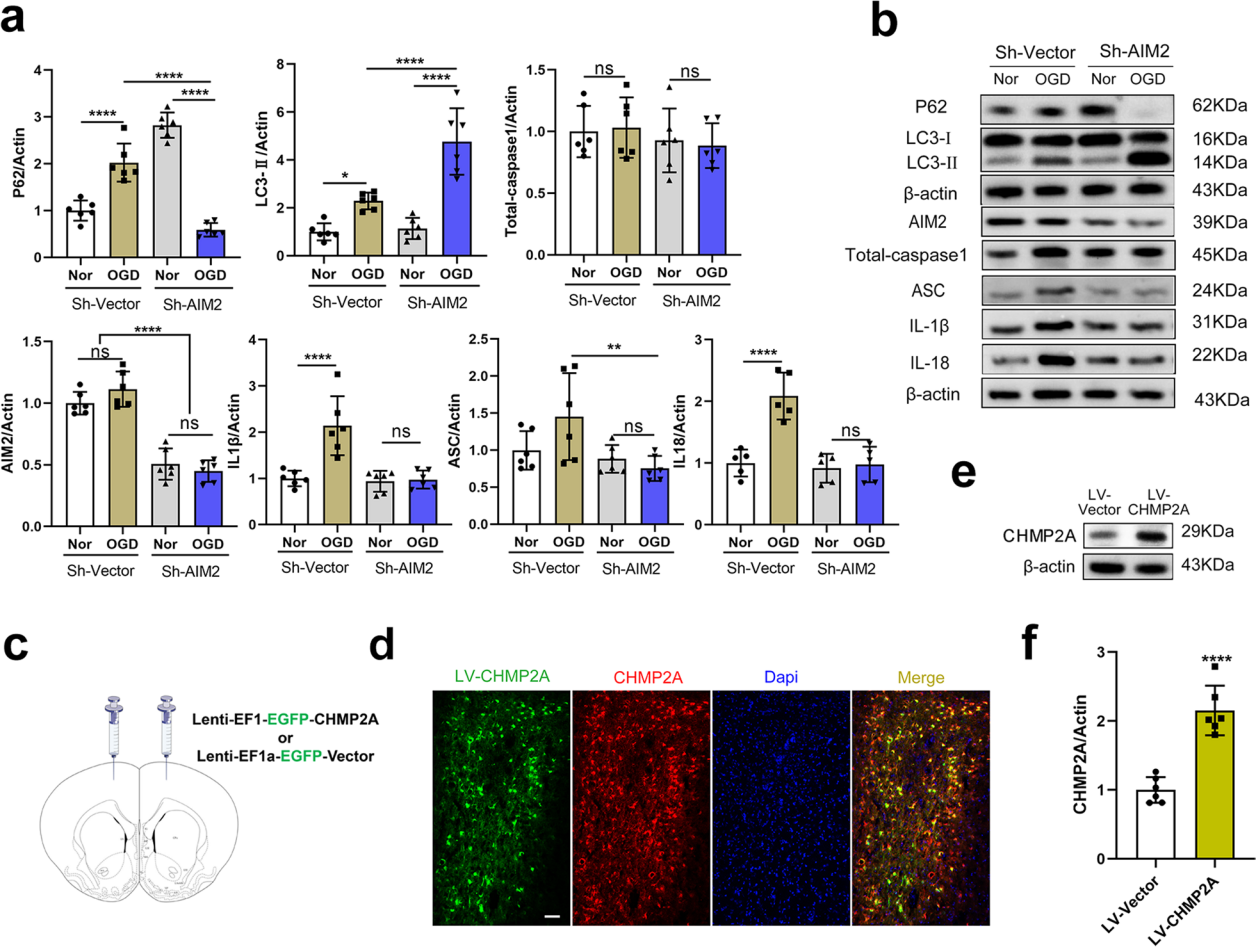
AIM2 knockdown abolishes OGD-induced autophagy damage and inflammation activation. **a**, **b** Representative Western blot and quantification of autophagy-related factors (LC3, p62) and AIM2-related factors (AIM2, total caspase-1, ASC, IL-1β, and IL-18) in PC12 cells infected with AIM2-specific shRNA (MOI = 20) or vector shRNA (MOI = 20) for 72 h and then subjected to OGD-Rep or normoxia (Nor) as a control. (*n* = 6, one-way ANOVA. **P < 0.01 and ****P < 0.0001). **c** Schematic showing that the Lenti-*EGFP* vector or Lenti-*EGFP*-*CHMP2A* virus was injected into the motor cortex. **d** Double immunofluorescence images of green fluorescence and CHMP2A protein (red) in the motor cortex on day 7 after Lenti-*EGFP-CHMP2A* virus injection. Scale bar, 1000 μm. **e**, **f** CHMP2A protein levels were elevated by LV-CHMP2A. (*n* = 5, Student’s *t* test. ****P < 0.0001 vs. LV-Vector group)

Researchers have not determined whether inflammation is the result or cause of impaired autophagic flux. On the one hand, when autophagy is blocked, the release of IL-1β is augmented, and inflammasomes are activated [34]. On the other hand, AIM2 inflammasome activation triggers caspase-1 activation, which inhibits mitophagy to amplify mitochondrial damage and pyroptotic cell death [35]. These findings led us to hypothesize that AIM2 may negatively regulate autophagy. Under normal circumstances, a reduction in AIM2 did not affect LC3 expression. After exposure to OGD, silencing AIM2 restored autophagic flux, as indicated by a dramatic increase in LC3-II and a rapid decrease in p62 levels (both *P* < 0.0001) (Fig. 6a, b). In addition, the inflammatory effect of OGD-Rep-induced AIM2 inflammasome activation, including IL-1β and IL-18 release (Fig. 6a, b), in PC12 cells was significantly abolished by AIM2 knockdown. However, the protein levels of ASC and caspase-1 did not change. Likewise, Shi et al. showed that the AIM2 ligand poly(dA:dT) initiates autophagosome formation in a sensor-dependent but ASC/caspase-1-independent manner [27]. This finding suggests that the early stages of inflammasome activation may have a functional effect on autophagy, although the fundamental mechanism is not fully understood and warrants further study. These results collectively indicate that a reduction in AIM2 may abrogate OGD-induced autophagy damage and inflammation activation.

Although our findings indicated a protective role of the reduction in AIM2 in enhancing autophagy and mitigating OGD-Rep-induced inflammation, we could not rule out the possibility that the non-AIM2-dependent signaling pathway may be induced to compensate and confer these protective effects.

### CHMP2A overexpression prevents neuroinflammation, protects against ischemic brain damage, and improves neurologic outcomes after CA

**Fig. 7:**
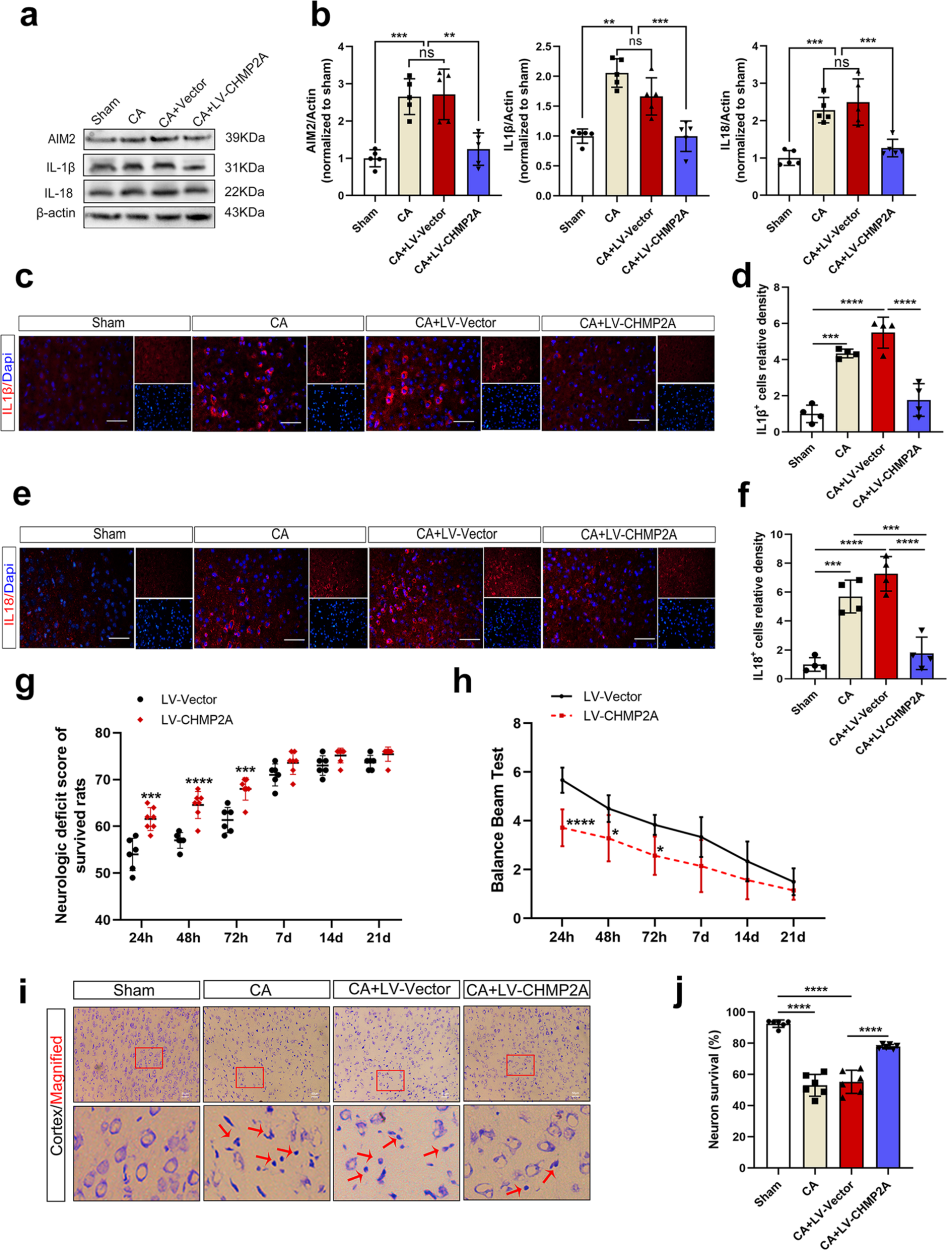
Overexpression of CHMP2A alleviates neuroinflammation and improves neurologic outcomes and neuropathological damage after CA. **a**, **b** Representative Western blot analyses of AIM2, IL-1β, and IL-18 protein levels in the motor cortex after exposure to CA-ROSC. **c**, **d** Representative immunofluorescence images and quantification of IL-1β (red) and DAPI (blue) double staining in the motor cortex of the sham, CA, CA+LV-Vector, and CA+LV-CHMP2A groups. Scale bar, 50 μm. **e**, **f** Representative immunofluorescence images and quantification of IL-18 (red) and DAPI (blue) double staining in the motor cortex of the sham, CA, CA+LV-Vector, and CA+LV-CHMP2A groups. Scale bar, 50 μm. **g** Scatter plots showing the difference in the distribution of NDSs (0 = brain death; 80 = normal) between the vector and CHMP2A groups. The data are presented as medians and interpercentile ranges (*n* = 6–7), two-factor (group × time) repeated-measures ANOVA. *P < 0.05, ***P < 0.001, and ****P < 0.0001. **h** Balance beam test scores of rats from each group. **i** Overexpression of CHMP2A decreased the number of Nissl-stained degenerative neurons in the motor cortex 72 h after CA-ROSC. Scale bar, 50 μm. The small red squares in the coronal section of the brain indicate the area observed. The red arrowheads indicate degenerative neurons with condensed staining and shrunken cytoplasm. **j** Quantification of Nissl-stained neuronal survival in the motor cortex. The data are expressed as means ± SEM (*n* = 4–6), one-way ANOVA. **P < 0.01, ***P < 0.001, and ****P < 0.0001 vs. sham

To further verify whether CHMP2A overexpression ameliorates neuroinflammation in the cortex after CA-ROSC, we injected LV-Vector or LV-CHMP2A virus into the motor cortex with stereotaxic coordinates and divided the animals into four groups: Sham, CA, CA+LV-Vector, and CA+LV-CHMP2A (*n* = 6–7 per group) (Fig. 6c). Efficient overexpression was verified by Western blotting and immunofluorescence staining (*P* < 0.0001, Fig. 6d–f). CHMP2A overexpression significantly decreased the protein levels of AIM2, IL-1β, and IL-18 in the cortex (*P* = 0.0011, *P* = 0.0025, and *P* = 0.0006, respectively) (Fig. 7a, b). These results were further confirmed by immunofluorescence staining, which showed dramatic reductions in IL-1β and IL-18 compared with those in the vector controls (both *P* < 0.0001) (Fig. 7c–f). These findings suggest that CHMP2A overexpression decreases AIM2 levels and thereby prevents the release of IL-1β and IL-18 in the brain after CA.

To assess whether the suppression of neuroinflammation by CHMP2A overexpression exerts neuroprotective effects on a rat model of CA, we used the NDSs and balance beam test to examine neurobehavioral impairments at 24 h, 48 h, 72 h, 7 days, 14 days, and 21 days after resuscitation. Compared with animals in the LV-Vector group, those injected with LV-CHMP2A exhibited significantly higher NDSs at 24 h (*P* < 0.001; Fig. 7g). The difference was still detectable at 72 h (*P* < 0.001) but diminished at 7 days (*P* > 0.05). In addition, a significantly lower balance beam test score was observed in the LV-Vector group than in the LV-CHMP2A group during the first 3 days after resuscitation (*P* < 0.05; Fig. 7h). The effect of CHMP2A overexpression on morphological changes in the motor cortex was further assessed. Nissl staining revealed that at 72 h after CA-ROSC, evidence of damaged neurons was visible, and these cells had shrunken nuclei and condensed staining compared with normal neurons. In addition, no significant difference was found between the CA and CA + vector groups (*P* = 0.8808; Fig. 7i). Quantitation of the number of viable neurons in the motor cortex suggested that CHMP2A overexpression increased the number of surviving neurons and protects against ischemic brain damage (*P* < 0.0001; Fig. 7j). Collectively, these data suggest that CHMP2A overexpression may ameliorate neurobehavioral impairment and improve neurologic outcomes after CA-ROSC.

## Discussion

**Fig. 8:**
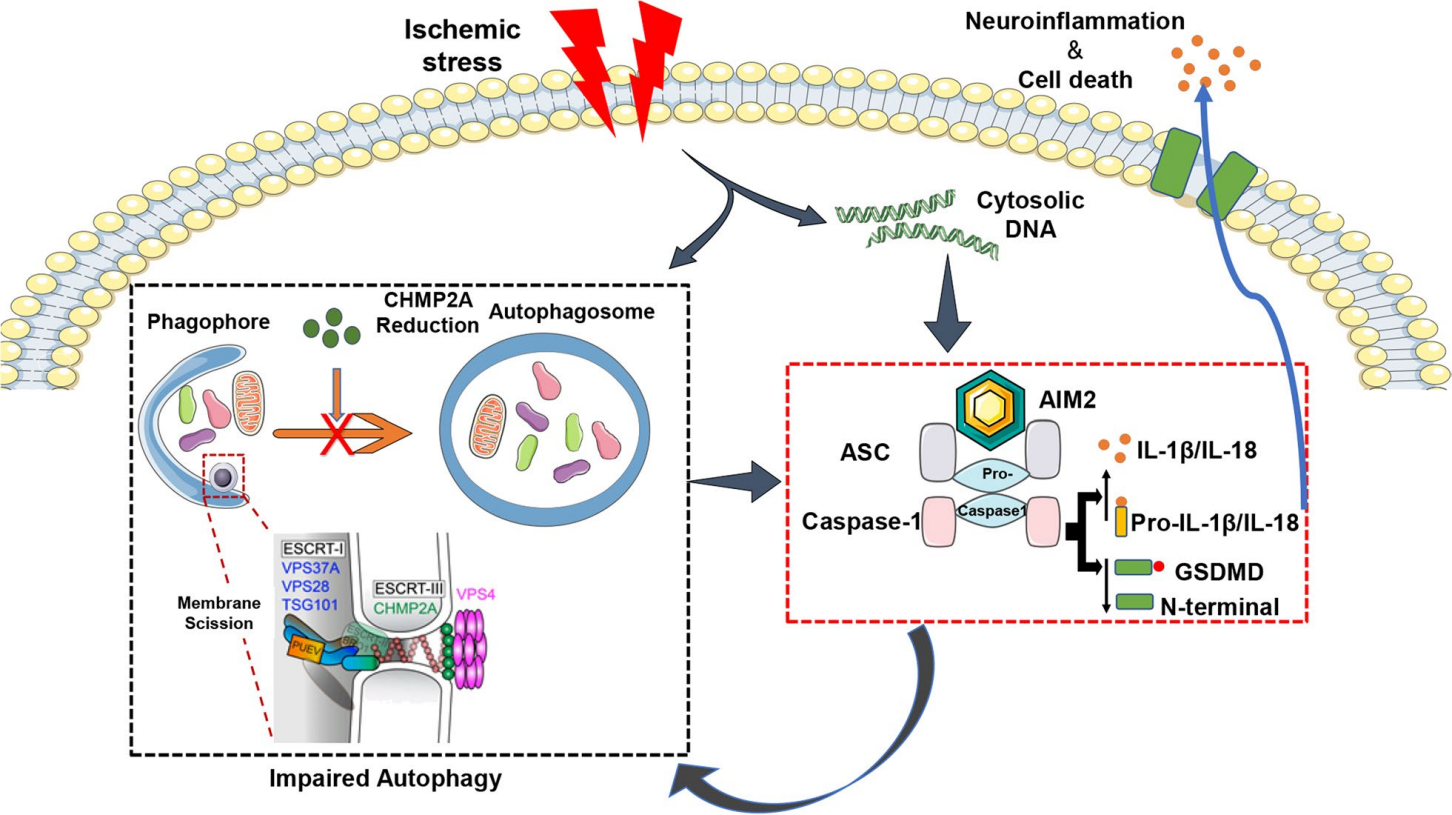
Schematic representation of AIM2-induced neuroinflammation and CHMP2A-mediated autophagy in CA-ROSC. Ischemia/reperfusion injury activates the AIM2 inflammasome and decreases CHMP2A expression. Impaired autophagic flux caused by a reduction in CHMP2A damages AIM2 inflammasome degradation and then triggers excessive inflammation, including cellular pyroptosis, which in turn impairs autophagy. In addition, AIM2 knockdown alleviates neuroinflammation and enhances the autophagic process after OGD-Rep. CHMP2A overexpression inhibits neuroinflammation and improves neurologic outcomes in CA-ROSC

A systemic inflammatory state called postcardiac arrest syndrome is a common problem in survivors and is characterized by oxidative stress, neuronal injury, and organ dysfunction [36]. Notably, recent evidence has provided novel insight into the inflammatory complexes known as inflammasomes, which contribute to neuronal cell death [37–39]. However, the underlying mechanism leading to this effect under ischemic conditions remains unknown. In the present study, AIM2 inflammasome activation and increased inflammatory cytokine (IL-1β and IL-18) secretion paralleled CHMP2A-mediated impaired autophagic flux after exposure to CA-ROSC. A reduction in CHMP2A resulted in autophagosome accumulation and suppressed autophagosome clearance. Therefore, impaired autophagic flux decreases AIM2 inflammasome autophagic degradation and increases IL-1β and IL-18 release, contributing to neuronal pyroptotic cell death. Interestingly, restoring the CHMP2A levels or silencing AIM2 restores autophagic flux, attenuates neuroinflammation, and improves neurologic outcomes after CA-ROSC (Fig. 8).

Accumulating evidence has demonstrated that the AIM2 inflammasome plays a crucial role in cerebral ischemia. The formation of the AIM2 inflammasome results in caspase-1 activation; subsequently, high levels of proinflammatory cytokines are detected and are correlated with the severity of inflammation and cellular damage [23, 40]. Denes et al. reported that AIM2^−/−^ mice had reduced ischemic brain injury, identifying AIM2 as a key driver of sterile inflammatory responses in the brain [41]. Similarly, inhibiting AIM2 inflammasome activation or double-stranded DNA (a trigger of the AIM2 inflammasome) alleviates GSDMD-induced pyroptosis and ameliorates brain injury after ischemic stroke [5, 6]. In our study, the AIM2 inflammasome was prone to activation synchronized with the upregulation of inflammatory factors in the cortex after CA-ROSC. In addition, the AIM2 inflammasome seemed to be exclusively abundant in neurons under ischemic conditions in vivo [42], a finding that was consistent with ours. Interestingly, pharmacologic stimulation (Rapa) or inhibition (CQ) of autophagy influenced AIM2 inflammasome activation and IL-1β/IL-18 production in vitro, suggesting that inflammasome clearance was accompanied by autophagy. However, further studies are required to establish the role of autophagy in inflammasomes under ischemic conditions.

The relationship between the AIM2 inflammasome and autophagy may be complex. Several studies provide compelling evidence that AIM2 inflammasome degradation and IL-1β secretion are autophagy-dependent [7, 32]. However, autophagic flux is enhanced by AIM2 activation [43]. Simultaneous activation of the inflammasome and autophagosome assembly limits the activity of the inflammasome; eventually, cells return to the basal state by eliminating inflammasomes under limited insult. By contrast, AIM2 inflammasome activation was accompanied by impaired autophagic flux in our model, which was characterized by increased autophagosomes and p62 expression. To further explore the underlying mechanism, we examined the colocalization of AIM2 with LC3 and CHMP2A, and the results suggested that enhanced AIM2 inflammasome activation is associated with impaired autophagosome clearance 12 h after CA-ROSC. To some extent, it is reasonable to hypothesize that when autophagosomes are blocked in the context of severe inflammatory stimuli, autophagy severely worsens the inflammatory response and even triggers cellular pyroptosis.

The ESCRT machinery sorts ubiquitinated cargo into multivesicular bodies initially and extends to mediate “reverse topology” membrane fission in several biological processes [44, 45]. By screening the main proteins of ESCRT-III, we found that inadequate CHMP2A may cause the failure of autophagosome membrane closure, which affects autophagic flux. Next, we found dose-dependent CHMP2A overexpression in PC12 cells and observed an increase in LC3 puncta formation and a dramatic decrease in p62 in cells subjected to OGD-Rep. However, minimal CHMP2A overexpression did not abrogate autophagy impairment. Because our gene overexpression approach did not supply adequate CHMP2A, the residual protein in CHMP2A-overexpressing cells may be insufficient to induce phagophore closure in PC12 cells exposed to OGD [14]. Conversely, CHMP2A knockdown impairs autophagic flux in PC12 cells and triggers cell death at levels comparable to those of infection with vectors, suggesting that the underlying mechanism is likely ESCRT-mediated membrane abscission to generate the outer/inner autophagosomal membrane [13]. Furthermore, the depletion of CHMP2A decreased autophagy by stabilizing intracellular death-inducing signaling complexes on immature autophagosomal membranes and increasing apoptosis in vivo [14]. Importantly, with the recovery of intact autophagic flux, we found that the AIM2 inflammasome and its downstream inflammatory cytokines were alleviated in vivo and in vitro. In addition, treatment with different autophagy inhibitors also consistently reversed the CHMP2A overexpression-mediated effects in the presence of OGD-Rep, supporting the potential link between autophagy and the AIM2 signaling pathway. However, phagophore closure may also be regulated by a noncanonical ESCRT pathway. For example, the interaction between CHMP2A and CHMP4B proteins is required for HIV budding [46]. CHMP7 regulates nuclear envelope reformation independent of the canonical pathway by targeting and bridging molecules [47]. Thus, the underlying mechanism by which CHMP2A compensates for autophagy remains unclear and warrants further study.

Inflammasome signaling pathways can modulate the autophagic process necessary to prevent excessive and detrimental inflammation. The NACHT domain of inflammasome-forming NLRs physically interacts with Beclin1 and inhibits the induction of autophagy [48]. NLRC4/caspase-1-deficient macrophages showed increased autophagosomes in response to Shigella infection [49]. Likewise, AIM2 enhances the induction of autophagy and inhibits the migration and invasion of renal carcinoma cells [18]. Interestingly, AIM2-dependent inflammasome activation is initially triggered by the HMGB1-DNA complex and subsequently stimulates ATG5-dependent autophagy, which acts as a negative-feedback loop for initial inflammasome activation [50]. To determine whether AIM2 knockdown could alleviate neuroinflammation and improve the autophagic process after OGD-Rep, we silenced AIM2 in PC12 cells exposed to OGD and evaluated the protein levels of autophagy-related factors and inflammasome-related factors. According to our results, a decline in AIM2 may inhibit OGD-induced autophagy damage and inflammation activation. These data provide new insights that AIM2 may negatively regulate autophagy during ischemic conditions.

There are some limitations for consideration when concluding this study. First, although a recent study reported that AIM2 specifically interacts with p62, instead of the proteins in initiation and elongation of autophagy [7], the molecular mechanism by which AIM2 complex regulates autophagy is still unclear and warrants further study. Second, we cannot exclude the possibility that the non-AIM2-dependent signaling pathway may be induced to compensate for the impaired autophagy flux, such as the NLRC4-dependent way in infection-induced autophagy [51] or the NLRP3 inflammasome–autophagy crosstalk [52]. Third, recent reports have shown that interactions among different cell types (neuron, astrocyte, and microglia) in neuroinflammation are paid more attention [53, 54], we cannot rule out the roles of other cells during cerebral ischemia–reperfusion injury. Further investigation is needed to elucidate the underlying mechanisms above these questions. Briefly, our findings provide a novel strategy for tuning the balance between AIM2-associated inflammation and autophagy activation to maintain neuroprotection during cerebral ischemia.

## Conclusions

In summary, our current study is the first to demonstrate that CHMP2A overexpression in a postcardiac arrest rat model exerts a neuroprotective role by decreasing neuroinflammation, improving neurological outcomes, and reducing neuronal loss in CA-ROSC rats. In addition, CHMP2A overexpression hinders the activation of the AIM2 inflammasome and production of proinflammatory mediators by restoring impaired autophagic flux in vitro. Interestingly, a decline in AIM2-associated inflammation may restore autophagic flux and inhibit inflammation activation. These findings provide clues concerning balancing inflammasome and autophagy activation during cerebral ischemia–reperfusion injury, in which regulating these pathways is a promising target for the treatment of ischemic stroke.

## Supplementary Information

**Additional file 1: Figure 1.** Colocalization of AIM2, LC3, and CHMP2A in the cortex after CA-ROSC. **a** Colocalization of AIM2 (red) with LC3 (green). **b** Colocalization of AIM2 (red) with CHMP2A (green). **c** Quantification of AIM2-positive cells and LC3-positive cells. **d** AIM2-positive cells and CHMP2A-positive cells in the cortex of sham-operated and injured rats. Scale bar, 50 μm. (*n* = 5, Student’s *t* test. *P < 0.05, **P < 0.01, and ****P < 0.0001 vs. sham).

**Additional file 2: Figure 2.** OGD injury upregulates autophagy and impairs autophagosome processing in PC12 cells. **a** Representative Western blot of LC3 and SQSTM1/p62 protein levels in PC12 cells subjected to OGD-Rep insults. **b** Quantification of LC3 and SQSTM1/p62 in PC12 cell lysates. The data are expressed as means ± SEM (*n* = 5, one-way ANOVA. *P < 0.05, **P < 0.01, ***P < 0.001, and ****P < 0.0001 vs. control).

## Data Availability

The datasets used and/or analysed during the current study are available from the corresponding author on reasonable request.

## Abbreviations

3-MA: 3-Methyladenine
ACTB: Actin beta
ASC/PYCARD: PYD and CARD domain containing
AIM2: Absent in melanoma 2
BECN1/beclin 1: Autophagy-related
CARD: Caspase recruitment domain
CEP55: Centrosomal protein 55
CHMP2A: Charged multivesicular body protein 2A
CHMP2B: Charged multivesicular body protein 2B
CHMP3: Charged multivesicular body protein 3
CHMP7: Charged multivesicular body protein 7
CQ: Chloroquine
DAPI: 4′,6-Diamidino-2-phenylindole, dihydrochloride
ESCRT: Endosomal sorting complex required for transport
GFAP: Glial fibrillary acidic protein
GSDMD: Gasdermin D
Iba1: Ionized calcium binding adaptor molecule 1
IL-18: Interleukin-18
IL-1β: Interleukin-1 beta
MAP1LC3B/LC3B: Microtubule-associated protein 1 light chain 3 beta
NeuN: Neuronal nuclei
Rapa: Rapamycin
RNA: Ribonucleic acid
SQSTM1/p62: Sequestosome 1
SEM: Standard error of the mean
TEM: Transmission electron microscopy
