# Supplementary figures and images for: The balance between AIM2-associated inflammation and autophagy: the role of CHMP2A in brain injury after cardiac arrest
Source: J Neuroinflammation. 2021 Nov 5;18:257. doi: 10.1186/s12974-021-02307-8 (PMC8571899; doi:10.1186/s12974-021-02307-8)

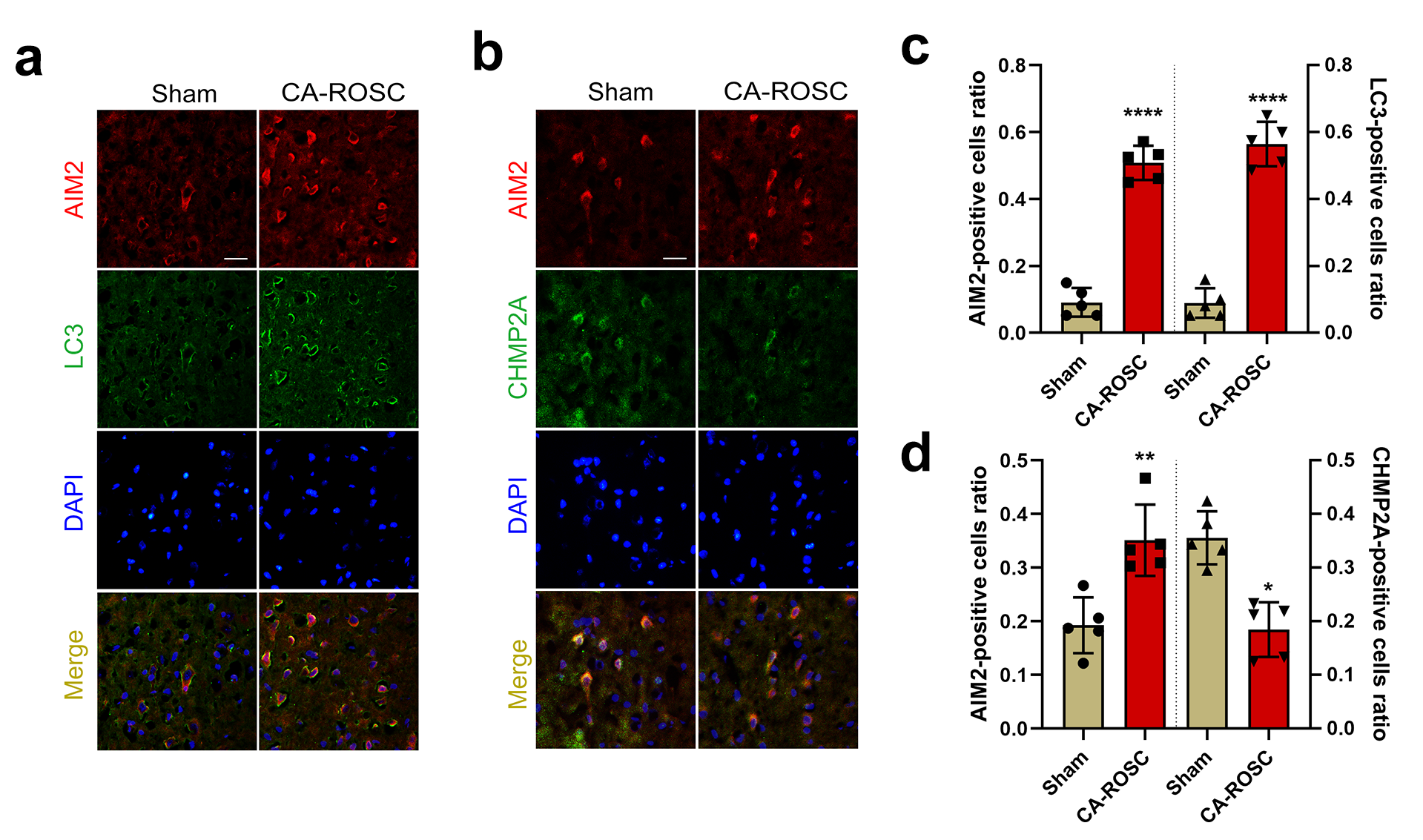

Supplement: Supplementary file 1 — Additional file 1: Figure 1. Colocalization of AIM2, LC3, and CHMP2A in the cortex after CA-ROSC. a Colocalization of AIM2 (red) with LC3 (green). b Colocalization of AIM2 (red) with CHMP2A (green). c Quantification of AIM2-positive cells and LC3-positive cells. d AIM2-positive cells and CHMP2A-positive cells in the cortex of sham-operated and injured rats. Scale bar, 50 μm. (n = 5, Student’s t test. *P < 0.05, **P < 0.01, and ****P < 0.0001 vs. sham). [file 12974_2021_2307_MOESM1_ESM.tif]

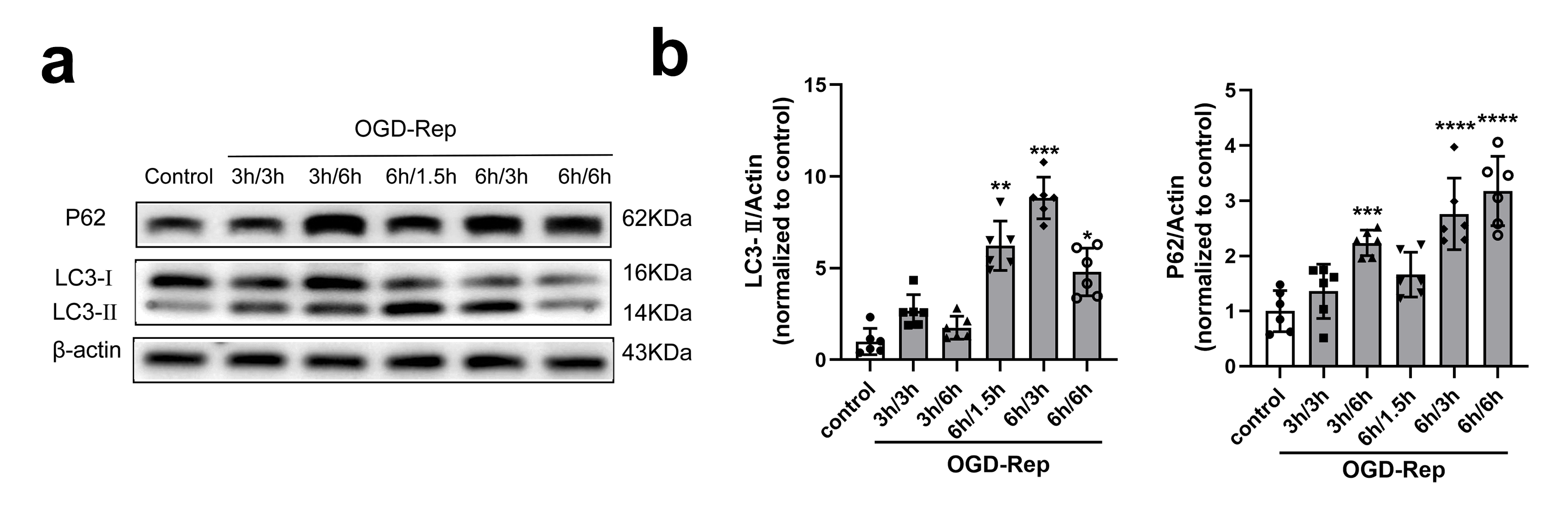

Supplement: Supplementary file 2 — Additional file 2: Figure 2. OGD injury upregulates autophagy and impairs autophagosome processing in PC12 cells. a Representative Western blot of LC3 and SQSTM1/p62 protein levels in PC12 cells subjected to OGD-Rep insults. b Quantification of LC3 and SQSTM1/p62 in PC12 cell lysates. The data are expressed as means ± SEM (n = 5, one-way ANOVA. *P < 0.05, **P < 0.01, ***P < 0.001, and ****P < 0.0001 vs. control). [file 12974_2021_2307_MOESM2_ESM.tif]
